# Alcance das Metas de Colesterol LDL após Infarto Agudo do Miocárdio: Dados Reais do Sistema Público de Saúde da Cidade de Curitiba

**DOI:** 10.36660/abc.20210328

**Published:** 2022-04-26

**Authors:** André Bernardi, Marcia Olandoski, Lucas Olandoski Erbano, Luiz Cesar Guarita-Souza, Cristina Pellegrino Baena, José Rocha Faria-Neto

**Affiliations:** 1 Faculdade de Medicina Pontifícia Universidade Católica do Paraná Curitiba PR Brasil Faculdade de Medicina, Pontifícia Universidade Católica do Paraná, Curitiba, PR – Brasil; 2 Hospital Universitário Evangélico Mackenzie Curitiba PR Brasil Hospital Universitário Evangélico Mackenzie, Curitiba, PR – Brasil

**Keywords:** Doenças Cardiovaculares, Infarto do Miocárdio, Dislipidemias, Prevenção Secundária, Diabetes Mellitus, Colesterol LDL, Epidemiologia, Prevenção e Controle, Fatores de Risco

## Abstract

**Fundamento:**

A redução dos níveis de colesterol LDL é a pedra angular na redução de risco, mas muitos pacientes de alto risco não estão atingindo as metas lipídicas recomendadas, mesmo em países de alta renda.

**Objetivo:**

Avaliar se os pacientes atendidos na rede pública de saúde da cidade de Curitiba estão atingindo as metas de colesterol LDL após infarto agudo do miocárdio (IAM).

**Métodos:**

Esta coorte retrospectiva explorou os dados de pacientes internados com IAM entre 2008 e 2015 em hospitais públicos da cidade de Curitiba. Para avaliar o atingimento da meta de colesterol LDL, utilizamos o último valor registrado no banco de dados para cada paciente até o ano de 2016. Para aqueles que tinham pelo menos um valor de colesterol LDL registrado no ano anterior ao IAM, calculou-se o percentual de redução. O nível de significância adotado para a análise estatística foi p<0,05.

**Resultados:**

Dos 7.066 pacientes internados por IAM, 1.451 foram acompanhados em ambiente ambulatorial e tiveram pelo menos uma avaliação de colesterol LDL. A média de idade foi 60,8±11,4 anos e 35,8%, 35,2%, 21,5% e 7,4% dos pacientes apresentavam níveis de colesterol LDL≥100, 70–99, 50–69 e <50 mg/dL, respectivamente. Destes, 377 pacientes também tiveram pelo menos uma avaliação de colesterol LDL antes do IAM. As concentrações médias de colesterol LDL foram 128,0 e 92,2 mg/dL antes e após o IAM, com redução média de 24,3% (35,7 mg/dL). Os níveis de colesterol LDL foram reduzidos em mais de 50% em apenas 18,3% dos casos.

**Conclusão:**

Na cidade de Curitiba, pacientes do sistema público de saúde, após infarto do miocárdio, não estão atingindo níveis adequados de colesterol LDL após IAM.

## Introdução

As doenças cardiovasculares (DCVs) são a principal causa de óbito no Brasil e no mundo. Globalmente, reportou-se uma estimativa de cerca de 18 milhões de óbitos por DCV em 2017, 85% das quais foram atribuídas a doenças isquêmicas do coração e cerebrovasculares.^1^ De acordo com as Estatísticas Cardiovasculares - Brasil, aproximadamente 388.268 pessoas morreram de DCV no país.^2^ Embora a taxa de mortalidade por doença isquêmica do coração (DIC) tenha permanecido estável na década de 2000,^3^ dados atuais mostram que a taxa de mortalidade por DIC padronizada por idade tem diminuído no Brasil.^2^

Níveis plasmáticos elevados de colesterol LDL (lipoproteína de baixa densidade) estão intimamente relacionados com o aumento do risco cardiovascular, independentemente da faixa etária.^4^ Além disso, a redução do colesterol LDL está associada à redução do risco cardiovascular: uma redução de 39 mg/dL está associada a uma redução de aproximadamente 20% no risco de eventos cardiovasculares maiores,^5^ um efeito semelhante entre os sexos.^6^ Em pacientes com alto risco de eventos cardiovasculares, principalmente aqueles com doença coronariana estabelecida, grandes reduções no Colesterol LDL com maiores doses de estatinas têm mostrado resultados melhores do que aquelas com doses inferiores.^7 , 8^ Da mesma forma, reduções adicionais no colesterol LDL, por meio do emprego de terapias adicionais combinadas com estatinas em pacientes de alto risco nas doses máximas otimizadas também estão associadas a reduções adicionais em novos eventos.^9 , 10^

Embora não se tenha identificado um nível mínimo ideal de colesterol LDL, sem risco de DCVs, os consensos e diretrizes atuais buscam estabelecer metas lipídicas para orientar o atendimento médico individualizado.^11 - 13^ Essas metas podem ser expressas como valores-alvo absolutos de colesterol LDL ou como porcentagens mínimas de redução do colesterol LDL. No entanto, muitos pacientes de alto risco não estão atingindo as metas lipídicas recomendadas,^14^ mesmo sob terapia hipolipemiante.^15^ Este é um problema multifatorial que requer quantificação em contextos locais específicos para garantir a viabilidade local e a eficácia das soluções propostas.^16^ No Brasil, embora a saúde seja considerada um dever do Estado, o acesso a estatinas mais potentes é limitado no Sistema Único de Saúde (SUS), sistema público de saúde brasileiro que atende mais de 70% da população.^17^

Até o momento, alguns estudos de mundo real foram conduzidos no Brasil, mostrando que pacientes com risco cardiovascular estão atingindo as metas lipídicas recomendadas.^18 , 19^ O objetivo deste estudo foi determinar a porcentagem de pacientes no sistema público de saúde da cidade de Curitiba, Brasil, que atingiram as metas de colesterol LDL após internação por infarto agudo do miocárdio (IAM), incluindo tanto o alcance dos valores-alvo de colesterol LDL quanto o percentual de redução do colesterol LDL em relação aos níveis anteriores ao IAM.

## Métodos

Trata-se de um estudo de coorte retrospectivo realizado no banco de dados da Secretaria Municipal de Saúde (SMS) de Curitiba, contendo todas as informações dos pacientes internados na rede pública municipal de saúde, desde a entrada até a alta. O presente estudo foi aprovado pelo Comitê de Ética em Pesquisa (CEP) da SMS e pela instituição acadêmica envolvida.

A coorte de pacientes selecionada no banco de dados incluiu pacientes de ambos os sexos com 18 anos ou mais, internados em um hospital público da rede de saúde local com diagnóstico primário de IAM (código CID-I21) entre janeiro de 2008 e dezembro de 2015. Os resultados dos exames laboratoriais foram obtidos a partir de um segundo banco de dados e as identidades dos pacientes foram verificadas minuciosamente para evitar duplicação e discrepâncias. Casos duplicados e casos com discrepâncias foram excluídos. Pacientes sem pelo menos um valor de colesterol LDL registrado no ano seguinte ao IAM também foram excluídos. Realizou-se uma busca no banco de dados do laboratório para encontrar os pacientes (entre os pacientes incluídos, ou seja, aqueles com pelo menos um exame após o IAM) que também haviam feito pelo menos um exame de colesterol LDL no ano anterior ao IAM para calcular o percentual de redução.

### Avaliação do colesterol LDL

Com base na fórmula de Friedewald, obteve-se o último valor de colesterol LDL, registrado na base de dados após o IAM, ou seja, o mais distante da data do IAM, exceto para pacientes com triglicerídeos acima de 400 mg/dL. Foram determinadas as porcentagens de pacientes que alcançaram níveis médios de colesterol LDL <50, 50–69, 70–99 ou ≥100 mg/dL.

Para determinar o percentual de redução alcançado, pesquisou-se o banco de dados em busca de pacientes com pelo menos um exame de colesterol LDL no ano anterior ao IAM. Nos casos de pacientes com mais de um exame, utilizou-se o valor de colesterol LDL mais próximo ao evento agudo. O valor de colesterol LDL mais próximo ao IAM no ano anterior ao evento foi comparado ao último valor obtido após o IAM. As porcentagens de pacientes que alcançaram reduções do colesterol LDL de 50–100% ou <50% ou aumentos <50% ou 50–100% também foram determinadas.

### Análise Estatística

Realizou-se a análise estatística descritiva dos dados. Os resultados foram expressos como médias e desvios-padrão (variáveis quantitativas) ou como frequências e porcentagens (variáveis categóricas). Utilizou-se o teste t de Student pareado para comparar o colesterol LDL antes e depois do IAM. A normalidade dos dados foi analisada pelo teste de Kolmogorov-Smirnov. Definiu-se a significância estatística com p<0,05. Os dados foram analisados com o programa computacional IBM SPSS Statistics v.20.0. Armonk, NY: IBM Corp.

## Resultados

**Figura 1:**
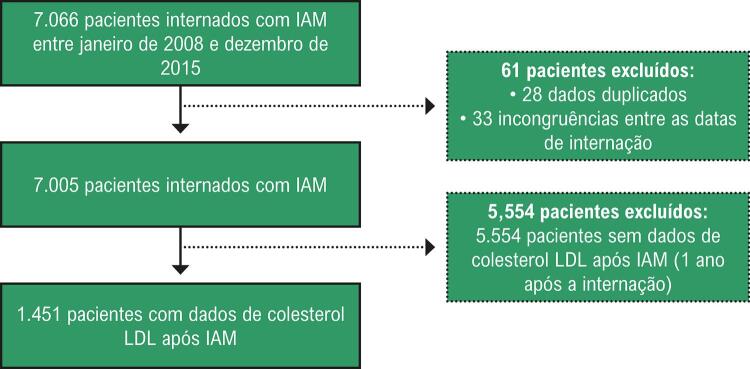
– Fluxograma das características da amostra do estudo. IAM: Infarto agudo do miocárdio; colesterol LDL: Colesterol LDL (lipoproteína de baixa densidade).

Do total de 7.066 pacientes internados por IAM entre janeiro de 2008 e dezembro de 2015, 61 foram excluídos devido a pelo menos um dos critérios de exclusão (duplicação ou discrepância entre as datas de internação). Dos 7.005 casos restantes, 5.554 foram excluídos por falta de resultados do exame de colesterol LDL após o IAM. Portanto, avaliou-se o colesterol LDL após o IAM em 1.451 casos ( Figura 1 ). Destes, 377 pacientes também realizaram pelo menos um exame no ano anterior ao IAM, o que permitiu o cálculo da variação percentual.

**Figura 2:**
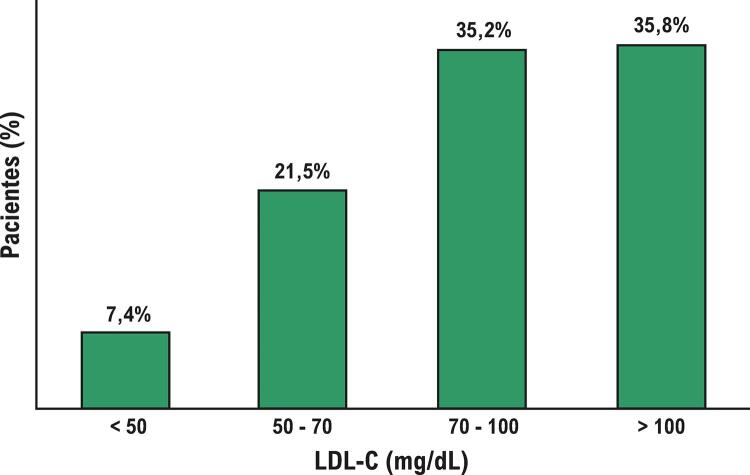
– Distribuição dos níveis de colesterol LDL (n=1.451). LDL-C: colesterol lipoproteína de baixa densidade.

A média de idade dos 1.451 pacientes foi 60,8±11,4. A Tabela 1 mostra a média e o desvio padrão (DP) do colesterol LDL entre os 1.451 casos após o IAM. O tempo médio até o último exame de colesterol LDL realizado após o IAM foi de 32,7 meses. A Figura 2 mostra as porcentagens dos níveis de colesterol LDL dos pacientes. Assim, apenas 28,9% dos pacientes apresentaram níveis de colesterol LDL <70 mg/dL após IAM.

**Tabela 1:** – Média e desvio padrão do colesterol LDL, colesterol HDL, colesterol total e triglicerídeos entre os 1.451 casos após o infarto agudo do miocárdio

	Média	DP
Colesterol LDL (mg/dL)	93,3	34,2
Colesterol HDL (mg/dL)	42,9	11,6
Colesterol total (mg/dL)	168,1	39,8

*LDL: lipoproteína de baixa densidade; HDL: lipoproteína de alta densidade; DP: desvio padrão.*

**Figura 3:**
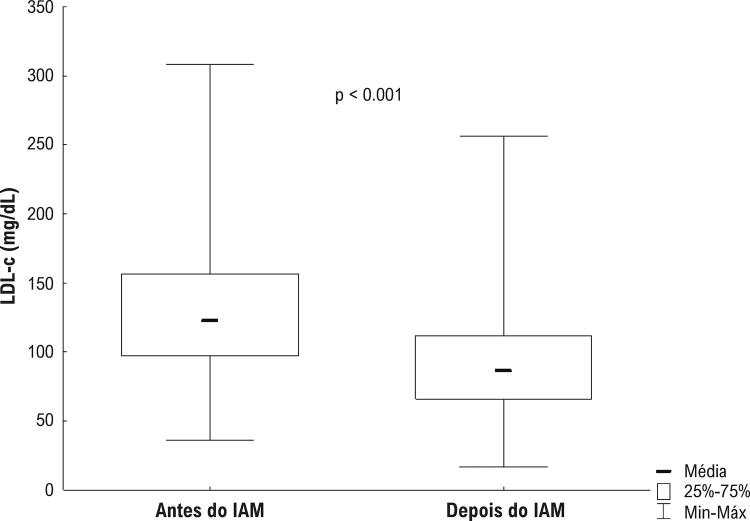
– Box-plot do colesterol LDL antes e depois do infarto agudo do miocárdio. Teste t de Student: p<0.05. IAM: Infarto agudo do miocárdio; colesterol LDL: Colesterol LDL (lipoproteína de baixa densidade).

**Figura 4:**
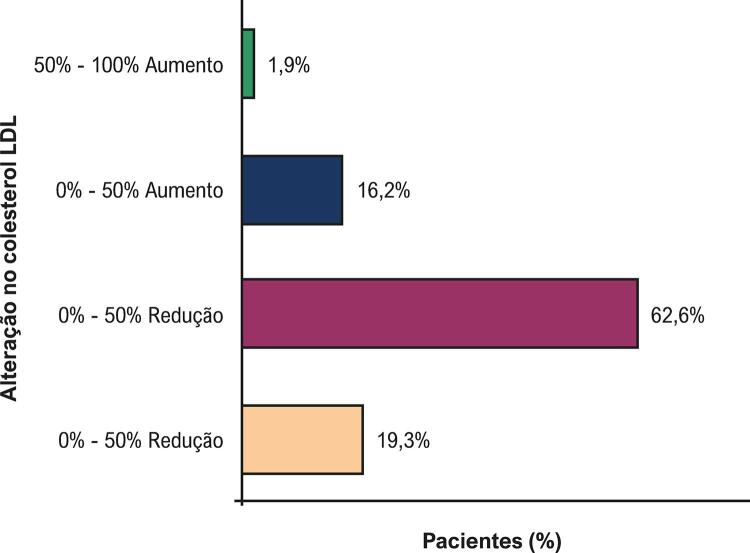
– Distribuição dos pacientes de acordo com a alteração no colesterol LDL antes e depois do infarto agudo do miocárdio.

Os valores de colesterol LDL após IAM, entre os 377 pacientes com dados de colesterol LDL no ano anterior ao IAM e pelo menos um exame de colesterol LDL após o evento, foram os seguintes: na mesma faixa de antes (40,3%), em uma faixa menor do que antes (53,3%), e em uma faixa maior do que antes (6,4%) ( Tabela 2 ). O tempo médio entre os exames de colesterol LDL antes e o mais próximo ao IAM e ao evento em si foi de 4,8 meses. As concentrações médias de colesterol LDL ( Figura 3 ) foram 128,0 e 92,2 mg/dL antes e após o IAM, respectivamente ( Tabela 3 ). A Figura 4 mostra que 19,3% dos pacientes tiveram uma redução de mais de 50% nos níveis de colesterol LDL após o IAM. Além disso, aproximadamente 82% dos pacientes alcançaram algum grau de redução do colesterol LDL ( Figura 4 ).

**Tabela 2:** – Distribuição dos níveis de colesterol LDL antes e depois do infarto agudo do miocárdio

Colesterol LDL após IAM (mg/dL)	Colesterol LDL antes do IAM (mg/dL)				Total

	<50	50–69	70–99	≥100	
<50	1	6	8	11	26
	0,3%	1,6%	2,1%	2,9%	
50–69	2	6	29	56	93
	0,5%	1,6%	7,7%	14,6%	
70–99	2	4	31	93	130
	0,5%	1,3%	8,2%	24,4%	
≥100	0	0	13	115	128
	0,0%	0,0%	3,7%	30,2%	
Total	6	17	82	272	377

*LDL: lipoproteína de baixa densidade; IAM: infarto agudo do miocárdio.*

**Tabela 3:** – Média e diminuição do colesterol de lipoproteína de baixa densidade antes e depois do infarto agudo do miocárdio entre os 377 casos

Variável	Média	DP	p*
Antes do IAM (mg/dL)	128,0	42,7	<0,001
Após IAM (mg/dL)	92,2	36,9	
Redução (absoluta) (mg/dL)	35,7	40,1	
Redução (relativa) (%)	24,3%	28,4%	

*Teste t de Student pareado, p<0,05. IAM: infarto agudo do miocárdio; DP: desvio padrão.

LDL: Colesterol LDL (lipoproteína de baixa densidade).

## Discussão

Apesar da eficácia da diminuição nas taxas de lipídios na redução de eventos cardiovasculares, muitos pacientes de alto risco não estão atingindo a meta lipídica recomendada. Este novo estudo realizado com dados de pacientes com IAM internados no sistema público de saúde de Curitiba constatou que aproximadamente 82% dos pacientes alcançaram algum grau de redução do colesterol LDL, com apenas aproximadamente 30% atingindo níveis médios <70 mg/dL e aproximadamente 20% tendo uma redução >50% em comparação com os níveis anteriores ao IAM.

Os resultados deste estudo são semelhantes aos conduzidos em contextos socioeconômicos muito diferentes. Dados recentes de 27 países europeus mostraram que, entre 8.261 pacientes coronarianos incluídos no estudo EUROASPIRE V, 80% estavam tomando estatinas e 71% tinham concentrações de colesterol LDL ≥70 mg/dL.^15^ Em um estudo americano mais antigo, que também avaliou pacientes após síndrome coronariana aguda (SCA) por meio da avaliação do controle lipídico no primeiro ano após o evento, apenas 31% dos pacientes atingiram o nível alvo de colesterol LDL <70 mg/dL.^20^ Os dados obtidos neste estudo são alarmantes por se tratarem de pacientes com pós-SCA, uma população de alto risco para novos eventos cardiovasculares a curto e médio prazo. O registro GRACE mostrou que aproximadamente 10% dos pacientes que receberam alta após SCA sofrerão IAM não fatal ou morte relacionada ao sistema cardiovascular em seis meses.^21^ Uma subanálise mais recente de pacientes com IAM prévio incluídos no estudo FOURIER demonstrou que um IAM mais recente apresenta maior risco de um novo evento cardiovascular do que um IAM mais distante (mais de dois anos) e esses pacientes são justamente aqueles que se beneficiam de uma redução lipídica mais agressiva.^22^

As metas propostas para os níveis de colesterol LDL foram extrapoladas a partir dos resultados de estudos com doses fixas de estatinas porque o primeiro estudo visando uma meta específica de colesterol LDL de 25–50 mg/dL foi conduzido apenas recentemente.^23^ Portanto, em 2013, a American Heart Association e o American College of Cardiology pararam de recomendar uma meta específica de colesterol LDL e propuseram o tratamento de pacientes de alto risco com altas doses de estatinas potentes capazes de reduzir o colesterol LDL em >50% com base nos resultados estudos intervencionais randomizados conduzidos nessas populações.^24^ Um estudo clínico que comparou estratégias para reduzir o risco cardiovascular (nível atingido ou porcentagem de redução) para determinar qual é o mais eficaz ainda não foi realizado, mas uma análise de dados de 13.937 pacientes dos três estudos distintos sobre prevenção secundária com estatinas sugere que uma redução de >50% reduziria o risco incrementalmente, mesmo em pacientes com níveis de colesterol LDL <70 mg/dL.^25^

Na presente amostra, mais pacientes alcançaram níveis de colesterol LDL <70 mg/dL do que aqueles que alcançaram uma redução de >50%. Isso pode ser explicado pelo fato de o percentual de redução estar diretamente associado ao uso de altas doses de estatinas potentes. O acesso a esses medicamentos no sistema público de saúde brasileiro é restrito e a indisponibilidade desses medicamentos neste sistema é uma barreira reconhecida ao seu uso.^26^ Há relatos de menor uso de medicamentos necessários para a prevenção secundária em países de baixa renda. Por exemplo, o estudo PURE relatou 66,5% e 3,3% do uso de estatinas para prevenção secundária em países de alta e baixa renda, respectivamente.^27^

Na época em que este estudo foi realizado, a 5ª Diretriz Brasileira de Dislipidemia e Prevenção da Aterosclerose^28^ recomendava metas de colesterol LDL abaixo de 70 mg/dL para pacientes com alto risco cardiovascular. Além disso, a recomendação para reduzir o colesterol LDL em pelo menos 50% aparece apenas na diretriz brasileira de 2017.^11^ As evidências atuais indicam que o benefício clínico não depende do tipo de estatina usada, mas sim da extensão da redução do colesterol LDL. Mais importante ainda é avaliar o risco cardiovascular do paciente e iniciar o tratamento visando a redução adequada do risco. Para pessoas de risco muito alto, uma meta de colesterol LDL de <55 mg/dL e uma redução de ≥50% do colesterol LDL basal deve ser alcançada.^13^

A American Association of Clinical Endocrinologists e o American College of Endocrinology propuseram uma meta de colesterol LDL de <55 mg/dL para uma nova categoria de risco denominada “risco extremo”.^29^ Esta categoria se refere a pacientes com doença cardiovascular aterosclerótica progressiva (DCAP), incluindo angina instável que persista mesmo após o alcance de um colesterol LDL de <70 mg/dL, ou DCAP clinicamente estável com diabetes, doença renal crônica estágio 3 ou 4 e/ou hipercolesterolemia familiar heterozigótica, ou pacientes com histórico de DCAP prematura (<55 anos de idade para homens ou <65 anos de idade para mulheres). Neste estudo, apenas 7,4% dos pacientes atingiram níveis inferiores a 50 mg/dL após IAM.

Embora as diretrizes americanas recomendem a redução dos níveis de colesterol LDL em pelo menos 50% do nível basal em pacientes coronarianos,^30^ as diretrizes europeias propõem uma meta de colesterol LDL de <55 mg/dL e uma redução mínima de 50% no colesterol LDL em pacientes com doença arterial coronariana (DAC) documentada.^13^ As diretrizes americanas e europeias recomendam o tratamento com uma combinação de medicamentos hipolipemiantes para atingir essas metas. No entanto, a diretriz americana concorda que o foco é a redução do colesterol LDL, principalmente com base em uma redução de >50% do valor basal, em vez de atingir os níveis-alvo específicos de colesterol LDL. No entanto, é importante destacar que os inibidores da pró-proteína convertase subtilisina/kexina tipo 9 (PCSK9) e a ezetimiba são aceitáveis em pacientes com IAM considerado de risco muito alto e com colesterol LDL ≥70 mg/dL em estatina com tolerância máxima.

Os resultados do estudo IMPROVE-IT mostraram que um número significativamente maior de pacientes com DAC tratados com estatina e ezetimiba alcançaram as metas de colesterol LDL em comparação com estatinas de maneira isolada.^31^

### Limitações do Estudo

Esta análise tem várias limitações possíveis. Apenas uma minoria dos pacientes internados com IAM na rede pública de saúde de Curitiba realizou exame de colesterol no ano seguinte ao IAM. Muitos pacientes que receberam tratamento em Curitiba provavelmente não eram da cidade. Portanto, a perda de seguimento ambulatorial foi significativa, pois esses pacientes retornaram às suas cidades de origem para acompanhamento médico e cuidados de prevenção secundária ou até mesmo interromperam o acompanhamento. Não foram obtidos dados sobre o colesterol LDL de pacientes que não receberam seguimento ambulatorial na rede pública de saúde de Curitiba. No entanto, a coorte de análise foi representativa de uma população real de Curitiba com infarto do miocárdio que sobreviveu à hospitalização. Por fim, a maior limitação deste estudo foi a ausência de dados sociodemográficos e medicamentosos, seja quanto ao uso (ou não) de estatinas, seja quanto às doses administradas antes e após o IAM.

## Conclusão

Após o IAM, uma minoria de pacientes de alto risco cardiovascular atingiu as metas de colesterol LDL recomendadas nesta coorte de pacientes internados no sistema público de saúde da cidade de Curitiba. A semelhança entre os resultados deste estudo e os de estudos realizados em países com condições socioeconômicas muito diferentes sugere que outros fatores, provavelmente relacionados aos próprios médicos e pacientes, podem estar associados a esse cenário.

Concepção e desenho da pesquisa: Bernardi A, Erbano LO, Guarita-Souza LC, Baena CP, Faria-Neto JR; Obtenção de dados: Bernardi A, Erbano LO; Análise e interpretação dos dados e Revisão crítica do manuscrito quanto ao conteúdo intelectual importante: Bernardi A, Olandoski M, Guarita-Souza LC, Baena CP, Faria-Neto JR; Análise estatística: Olandoski M, Erbano LO, Faria-Neto JR; Redação do manuscrito: Bernardi A, Faria-Neto JR.
