# Supplementary material for: Alcance das Metas de Colesterol LDL após Infarto Agudo do Miocárdio: Dados Reais do Sistema Público de Saúde da Cidade de Curitiba
Source: Arq Bras Cardiol. 2022 Apr 26;118(6):1018–25. [Article in Portuguese] doi: 10.36660/abc.20210328 (PMC9345156; doi:10.36660/abc.20210328)
Supplement: Translation

# Translation: Achievement of LDL-Cholesterol Goals after Acute Myocardial Infarction: Real-World Data from the City of Curitiba Public Health System

**Authors:** André Bernardi, Marcia Olandoski, Lucas Olandoski Erbano, Luiz Cesar Guarita-Souza, Cristina Pellegrino Baena, José Rocha Faria-Neto

**Affiliations:** 1 Brazil Faculdade de Medicina, Pontifícia Universidade Católica do Paraná, Curitiba, PR – Brazil; 2 Brazil Hospital Universitário Evangélico Mackenzie, Curitiba, PR – Brazil

**Keywords:** Cardiovascular Diseases, Myocardial Infarction, Dyslipidemias, Secondary Prevention, Diabetes Mellitus, Choleterol LDL, Epidemiology, Prevention and Control, Risk Factors

## Abstract

**Background:**

Reduction of LDL-cholesterol (LDL-c) levels is the cornerstone in risk reduction, but many high-risk patients are not achieving the recommended lipid goals, even in high-income countries.

**Objective:**

To evaluate whether patients seen in the city of Curitiba public health system are reaching LDL-c goals after an acute myocardial infarction (AMI).

**Methods:**

This retrospective cohort explored the data of patients admitted with AMI between 2008 and 2015 in public hospitals from the city of Curitiba. In order to evaluate the attainment of the LDL-c target, we have used the last value registered in the database for each patient up to 2016. For those who had at least one LDL-c registered in the year before AMI, percentage of reduction was calculated. The level of significance adopted for statistical analysis was p<0.05.

**Results:**

Of 7,066 patients admitted for AMI, 1,451 were followed up in an out-patient setting and had at least one evaluation of LDL-c. Mean age was 60.8±11.4 years and 35.8%, 35.2%, 21.5%, and 7.4% of patients had LDL-c levels ≥100, 70–99, 50–69 and <50 mg/dL, respectively. Of these, 377 patients also had at least one LDL-c evaluation before the AMI. Mean LDL-c concentrations were 128.0 and 92.2 mg/dL before and after AMI, with a mean reduction of 24.3% (35.7 mg/dL). LDL-c levels were reduced by more than 50% in only 18.3% of the cases.

**Conclusion:**

In the city of Curitiba public health system patients, after myocardial infarction, are not achieving adequate LDL-c levels after AMI.

## Introduction

Cardiovascular diseases (CVDs) are the leading cause of death in Brazil and worldwide. Globally, it is estimated that there were 18 million deaths from CVDs in 2017, 85% of which were attributed to ischemic heart and cerebrovascular diseases.^1^According to the Cardiovascular Statistics – Brazil, approximately 388,268 people died from CVDs in this country.^2^Although the mortality rate for ischemic heart disease (IHD) remained stable in the 2000s,^3^current data have shown that age-standardized mortality rate from IHD has been decreasing in Brazil.^2^

High plasma low-density lipoprotein cholesterol (LDL-c) levels are closely correlated with increased cardiovascular risk, regardless of the age group.^4^Moreover, reduction in LDL-c is associated with reduced cardiovascular risk: a 39 mg/dL decrease is associated with an approximate 20% reduction in the risk of major cardiovascular events,^5^an effect that is similar between sexes.^6^In patients at high risk for cardiovascular events, especially those with established coronary disease, massive LDL-c reductions with higher doses of statins have shown better results than those for lower doses.^7 , 8^Similarly, additional reductions in LDL-c using additional therapies combined with statins in highest-risk patients at the optimized maximum doses are also associated with further reduction in new events.^9 , 10^

Although an optimal minimum LDL-c level at which there is no risk for CVDs has not been identified, the current consensuses and guidelines seek to establish lipid goals to guide individualized medical care.^11 - 13^These goals may be expressed as absolute LDL-c target values or as minimum percentages of LDL-c reduction. However, many high-risk patients are not achieving the recommended lipid goals,^14^even under lipid-lowering therapy.^15^This is a multifactorial problem requiring quantification in specific local contexts to ensure the local feasibility and effectiveness of the proposed solutions.^16^In Brazil, although health is considered a duty of the State, access to potent statins is limited in the Unified Health System (SUS), the Brazilian public health system that assists more than 70% of the population.^17^

Until now, a few real-world studies have been conducted in Brazil, showing that patients at cardiovascular risk are achieving the recommended lipid goals.^18 , 19^The objective of this study was to determine the percentage of patients in the public health system from the city of Curitiba, Brazil, who achieved the LDL-c goals after admission for acute myocardial infarction (AMI), including both the attainment of the target LDL-c level and the percentage of LDL-c reduction compared to the levels before AMI.

## Method

This retrospective cohort study was conducted using the Municipal Health Secretariat of Curitiba (SMS) database containing all information on patients admitted to the city’s public health system from the date of admission to the date of discharge. This study was approved by the SMS Research Ethics Committee (REC) and by the academic institution involved.

The patient cohort selected from the database included those of both sexes aged 18 and over, who were admitted to a local public hospital with primary diagnosis of AMI (code ICD-I21) between January 2008 and December 2015. The laboratory test results were obtained from a second database and patient IDs were thoroughly checked to avoid duplication and inconsistency. Duplicate cases and cases with inconsistencies were excluded. Patients without at least one LDL-c value recorded in the year following AMI were also excluded. A search was performed in the laboratory database to find those patients (among the included patients, i.e., those with at least one test after the AMI) who also had at least one LDL-c test in the year before the AMI to calculate the percentage reduction.

### LDL-c evaluation

The last LDL-c value, based on the Friedewald formula, recorded in the database following AMI, i.e., the most distant from the date of the AMI, was obtained, except for patients with triglycerides over 400 mg/dL. The percentages of patients who achieved mean LDL-c levels <50, 50–69, 70–99, or ≥100 mg/dL were determined.

To determine the percentage reduction achieved, the database was searched for patients with at least one LDL-c test in the year before the AMI. In cases of patients with more than one test, the LDL-c value closest to the acute event was used. The LDL-c value closest to the AMI in the year before the event was compared to the last value obtained after the AMI. The percentages of patients who achieved LDL-c reductions of 50–100% or <50% or with <50% or 50–100% increases were also determined.

### Statistical Analysis

A descriptive statistical analysis of the data was carried out. The results were expressed as means and standard deviations (quantitative variables) or as frequencies and percentages (categorical variables). Paired Student’s t-test was used to compare LDL-c before and after AMI. Data normality was analyzed by Kolmogorov-Smirnov test. Statistical significance was accepted for *p* <0.05. Data were analyzed using IBM SPSS Statistics v.20.0. Armonk, NY: IBM Corp.

## Results

Of 7,066 total patients admitted for AMI between January 2008 and December 2015, 61 were excluded due to at least one of the exclusion criteria (duplication or inconsistency in dates of admission). Of the 7,005 remaining cases, 5,554 were excluded for lack of LDL-c results after the AMI. Therefore, the level of LDL-c after the AMI event was evaluated in 1,451 cases ( Figure 1 ). Of these, 377 patients also had at least one test in the year before the AMI, which allowed calculation of the percentage variation.

**Figure 1:** – Flowchart of study sample characteristics. AMI: Acute myocardial infarction; LDL-c: Low-density lipoprotein cholesterol.

The mean age of the 1,451 patients was 60.8±11.4. Table 1 shows the mean and the standard deviation (SD) of LDL-c among the 1,451 cases after the AMI event. The mean time to the last LDL-c test performed after the AMI was 32.7 months. Figure 2 shows the patients’ percentages of LDL-c levels. Thus, only 28.9% of the patients had LDL-c levels <70 mg/dL after AMI.

**Table 1:** – Mean and standard deviation of low-density lipoprotein cholesterol, high-density lipoprotein cholesterol, total cholesterol and triglycerides among the 1,451 cases after acute myocardial infarction

	Mean	SD
LDL-c (mg/dL)	93.3	34.2
HDL-c (mg/dL)	42.9	11.6
Total cholesterol (mg/dL)	168.1	39.8

*LDL-c: Low-density lipoprotein cholesterol; HDL-c: High-density lipoprotein cholesterol; SD: standard deviation.*

**Figure 2:** – Distribution of low-density lipoprotein cholesterol (LDL-c) levels (n=1,451). LDL-c: Low-density lipoprotein cholesterol.

LDL-c values after AMI, among the 377 patients with LDL-c data in the year before the AMI and at least one LDL-c test after the event, were as follows: in the same range as before (40.3%), in a lower range than before (53.3%), and in a higher range than before (6.4%) ( Table 2 ). The mean time between the LDL-c tests before and closest to the AMI and the event itself was 4.8 months. The mean LDL-c concentrations ( Figure 3 ) were 128.0 and 92.2 mg/dL before and after AMI, respectively ( Table 3 ). Figure 4 shows that 19.3% of patients had a more than 50% reduction in LDL-c levels after AMI. Additionally, approximately 82% of the patients achieved some degree of LDL-c reduction ( Figure 4 ).

**Table 2:** – Distribution of low-density lipoprotein cholesterol levels before and after acute myocardial infarction

LDL-c after AMI (mg/dL)	LDL-c before AMI (mg/dL)				Total

	<50	50–69	70–99	≥100	
<50	1	6	8	11	26
	0.3%	1.6%	2.1%	2.9%	
50–69	2	6	29	56	93
	0.5%	1.6%	7.7%	14.6%	
70–99	2	4	31	93	130
	0.5%	1.3%	8.2%	24.4%	
≥100	0	0	13	115	128
	0.0%	0.0%	3.7%	30.2%	
Total	6	17	82	272	377

*LDL-c: Low-density lipoprotein cholesterol; AMI: acute myocardial infarction.*

**Figure 3:** – Box-plot for low-density lipoprotein before and after acute myocardial infarction. Student’s t-test, p<0.05. AMI: acute myocardial infarction;

LDL-c: Low-density lipoprotein cholesterol.

**Table 3:** – Mean and decrease in low-density lipoprotein cholesterol before and after acute myocardial infarction among the 377 cases

Variable	Mean	SD	p*
Before AMI (mg/dL)	128.0	42.7	<0.001
After AMI (mg/dL)	92.2	36.9	
Decrease (absolute) (mg/dL)	35.7	40.1	
Decrease (relative) (%)	24.3%	28.4%	

*Paired Student’s t test, p<0.05. AMI: acute myocardial infarction; SD: standard deviation.

**Figure 4:** – Distribution of patients according to the change in low-density lipoprotein cholesterol before and after acute myocardial infarction.

LDL-c: Low-density lipoprotein cholesterol.

## Discussion

Despite the effectiveness of lipid reduction on the reduction of cardiovascular events, many high-risk patients are not achieving the recommended lipid goal. This novel study conducted with data on AMI patients admitted to the public health system of Curitiba found that approximately 82% of the patients achieved some degree of LDL-c reduction, with only approximately 30% attaining mean levels <70 mg/dL and approximately 20% having a reduction >50% compared to the levels before AMI.

The results of this study are similar to those conducted in very different socioeconomic contexts. Recent data from 27 European countries showed that, among 8,261 coronary patients included in the EUROASPIRE V study, 80% were using statins and 71% had LDL-c concentrations ≥70 mg/dL.^15^In an older US study also evaluating patients after acute coronary syndrome (ACS) through assessment of lipid control in the first year after the event, only 31% of patients achieved the target LDL-c level <70 mg/dL.^20^The data obtained in this study are alarming because these are post-ACS patients, a population at very high risk for new cardiovascular events in the short- to medium-term. The GRACE Registry showed that approximately 10% of patients discharged after an ACS will suffer a non-fatal AMI or a cardiovascular-related death within six months.^21^A more recent subanalysis of patients with prior AMI included in the FOURIER study demonstrated that a more recent AMI presents a higher risk for a new cardiovascular event than a more distant AMI (more than two years) and these patients are precisely the ones who benefit from a more aggressive lipid reduction.^22^

The proposed goals for LDL-c levels were extrapolated from the results of studies with fixed doses of statins because the first study aiming at a specific LDL-c target of 25–50 mg/dL was only recently conducted.^23^Therefore, in 2013, the American Heart Association and the American College of Cardiology stopped recommending a specific LDL-c goal and proposed the treatment of high-risk patients with high doses of potent statins capable of reducing LDL-c by >50% based on the results of randomized intervention studies conducted in these populations.^24^A clinical study comparing strategies to reduce cardiovascular risk (level attained or percentage of reduction) to determine which is the most effective has not yet been performed, but an analysis of data on 13,937 patients from the three distinct studies on secondary prevention with statins suggests that a >50% reduction would reduce the risk incrementally, even in patients with LDL-c levels <70 mg/dL.^25^

In the present sample, more patients achieved LDL-c levels <70 mg/dL than those achieving a >50% reduction. This may be explained by the fact that the percentage of reduction is directly associated with the use of high-dose potent statins. Access to these medications within the Brazilian public health system is restricted and the unavailability of these medications in this system is a recognized barrier to their use.^26^Lower use of medications necessary for secondary prevention in lower-income countries has been reported. For instance, the PURE study reported 66.5% and 3.3% statin use for secondary prevention in high-and low-income countries, respectively.^27^

By the time this study was conducted, the 5^th^Brazilian Guideline on Dyslipidemia and Prevention of Atherosclerosis^28^recommended LDL-c goals under 70 mg/dL for patients with high cardiovascular risk. Moreover, the recommendation to lower LDL-c by at least 50% appears only in the 2017 Brazilian guideline.^11^Current evidence indicates that the clinical benefit does not depend on the type of statin used but rather on the extent of LDL-c reduction. Most importantly, it is necessary to assess the patient’s cardiovascular risk and initiate treatment aiming at adequate risk reduction. For very high-risk individuals, an LDL-c goal of <55 mg/dL and a reduction of ≥50% from baseline LDL-c should be achieved.^13^

The American Association of Clinical Endocrinologists and the American College of Endocrinology proposed an LDL-c goal of <55 mg/dL for a new category of risk termed “extreme risk”.^29^This category refers to patients with progressive atherosclerotic cardiovascular disease (ASCVD), including unstable angina persisting after an LDL-c of <70 mg/dL has been achieved, or clinically stable ASCVD with diabetes, stage 3 or 4 chronic kidney disease and/or heterozygous familial hypercholesterolemia, or patients with a history of premature ASCVD (<55 years of age for men or <65 years of age for women). In this study, only 7.4% of patients achieved levels lower than 50 mg/dL after AMI.

Whereas the American guidelines recommend lowering LDL-C levels by at least 50% of the baseline in coronary patients,^30^the European guidelines propose a target LDL-c of <55 mg/dL and at least a 50% reduction in LDL-c in patients with documented coronary artery disease (CAD).^13^The American and European guidelines recommend treatment with a combination of lipid-lowering drugs to achieve these goals. However, the American guideline agrees that the focus is LDL-c reduction, mainly based on a >50% reduction from the baseline value rather than on the attainment of specific LDL-c target levels. However, it is important to highlight that proprotein convertase subtilisin/kexin type 9 (PCSK9) inhibitors and ezetimibe are reasonable in patients with AMI considered to be at very high risk and with LDL-c ≥ 70 mg/dL on maximally tolerated statins.

The results of the IMPROVE-IT study showed that significantly more patients with CAD treated with a combination of statin and ezetimibe achieved the LDL-c goals compared to statins alone.^31^

### Study limitations

This analysis has several potential limitations. Only a minority of patients admitted for AMI in the public health system of Curitiba underwent a cholesterol test in the year after the AMI. Many patients that were treated for the event in Curitiba were likely not actually from the city. Therefore, the loss to outpatient follow-up was significant because these patients returned to their hometowns for medical follow-up and secondary prevention care or even discontinued follow-up care. No LDL-c data from patients who did not receive outpatient follow-up in the public health system of Curitiba were obtained. Nevertheless, the analysis cohort was representative of a real-world population of Curitiba with myocardial infarction that survived hospitalization. Lastly, the greatest limitation of this study was the absence of sociodemographic and medication details, either regarding the use (or not) of statins or the doses administered before and after AMI.

## Conclusion

After AMI, a minority of cardiovascular high-risk patients achieved the recommended LDL-c goals in this cohort of patients admitted to the city of Curitiba public health system. The similarity between the results of this study and those from studies conducted in countries with very different socioeconomic conditions suggests that other factors, probably related to physicians and patients themselves, may be associated with this scenario.
